# Feasibility of a web video-based positive word stimulation program for older patients with cardiac disease and subthreshold depression admitted to an acute care ward: a pilot randomized controlled trial

**DOI:** 10.1007/s41999-025-01258-0

**Published:** 2025-06-23

**Authors:** Masataka Sakimoto, Hiroyuki Uchida, Takumi Igusa, Takuya Kobayashi, Aya Fukazawa, Chihaya Machida, Hirokuni Fujii, Keisuke Sekine, Minori Kurosaki, Kenji Tsuchiya, Senichiro Kikuchi, Kazuki Hirao

**Affiliations:** 1 Graduate School of Health Sciences, Gunma University, Maebashi, Japan; 2 Department of Rehabilitation, Fujioka General Hospital, Fujioka, Japan; 3 Department of Rehabilitation, Kurashiki Heisei Hospital, Kurashiki, Japan; 4 Department of Rehabilitation, Faculty of Health Sciences, Nagano University of Health Medicine, Nagano, Japan; 5 Department of Occupational Therapy, School of Health Sciences, Faculty of Medicine, Kagoshima University, 8-35-1 Sakuragaoka, Kagoshima, 890-8544 Japan

**Keywords:** Subthreshold depression, Cardiac disease, Older adults, Mental health

## Abstract

**Purpose:**

Major depressive disorder can exacerbate morbidity and hasten mortality from cardiac disorders among geriatric patients, so mitigation of subthreshold depression (StD) may help improve long-term outcome. Subliminal priming with supraliminal reward stimulation (SPSRS), in which subjects view videos with embedded positive words, may be an effective intervention strategy for StD symptom reduction. The purpose of this pilot randomized controlled trial (RCT) was to evaluate the feasibility of an SPSRS intervention for older patients with cardiac disease and StD admitted to acute care wards.

**Methods:**

Thirty older patients with cardiac disease and comorbid StD (79.2 ± 7.55 years, six female) were randomly assigned to the intervention (SPSRS) group (*n* = 15) or the control group (*n* = 15). The intervention group viewed 10-min videos containing positive word stimulation once per day for 5 consecutive days, while the control group viewed YouTube videos containing no positive word stimulation following the same protocol. The primary outcome was a change in score on the Japanese version of the Beck Depression Inventory-II (BDI-II).

**Results:**

There were no dropouts and no adverse events during the trial. Linear mixed models showed no significant improvement in BDI-II in the intervention group compared to the control group (*P* = 0.72).

**Conclusions:**

This pilot RCT supports the feasibility of the SPSRS intervention for older patients with cardiac disease and StD. A full-scale RCT would require a larger sample size, modifications to the content for this patient group, increased frequency of daily interventions, and a more sensitive depression symptom assessment tool.

Trial registration: The University Hospital Medical Information Network (UMIN) Registered 8 September 2023 (UMIN000052155).

## Key summary points

**Aim:**

This pilot randomized controlled trial (RCT) evaluated the feasibility of a web-based subliminal priming and supraliminal reward stimulation (SPSRS) intervention for mitigating depressive symptoms in older patients with cardiac disease and subthreshold depression (StD) hospitalized in an acute care ward.

**Findings:**

The SPSRS intervention did not cause any adverse events but depressive symptoms were not significantly improved in the intervention group compared to the control group.

**Message:**

Possible reasons for this lack of antidepressant efficacy include limited statistical power due to the small sample size, insufficient intervention frequency and duration, inappropriate content (specific words presented) for the target population, and inadequate sensitivity of the outcome indicators. A full-scale RCT is not feasible until these factors are modified for the target population.

## Introduction

Major depressive disorder (MDD) is major contributor to the total global burden of disease attributed by cardiac disorders [1]. It has been suggested that comorbid MDD can increase the risks of serious cardiovascular events, reduce quality of life, and escalate health care costs among cardiac disorder patients, ultimately leading to more severe morbidity and earlier mortality [2–12]. Previous studies have reported that 15%–20% of cardiac disease patients requiring hospitalization have comorbid MDD, a prevalence about twice that of the general population [13–15]. This association may be further exacerbated by population aging [5, 9, 16–19]. Treating MDD directly can help alleviate the combined burden of MDD and cardiac disorders in older adults [20–22]. However, approximately one-third of all patients diagnosed with MDD do not respond to current treatments [23], and response to treatment may be even lower [24–27]. Therefore, interventions for preventing MDD occurrence in older adults with cardiac disease are crucial, and subthreshold depression (StD) is a significant target [28–33].

StD does not meet the diagnostic criteria for MDD; however, it is characterized by clinically significant depressive symptoms and is a risk factor for future MDD [34–37]. Previous studies have suggested that StD prevalence is not only on the rise among the older population [38, 39], but is even more common among patients with cardiac disease [40–42]. In addition to exacerbating cardiac disease-related morbidity and mortality, StD alone presents a significant clinical burden [34, 43–49]. Given the seriousness of StD to older patients both with and without cardiac disease, it is important to establish safe and cost-effect prevention and mitigation strategies. Numerous preventive intervention strategies are available for improving depressive symptoms among older adults with cardiovascular diseases and other comorbidities, including antidepressant drugs (ADDs), cognitive behavioral therapy (CBT), and exercise therapy [49–52]. However, all present challenges for antidepressant treatment of the geriatric cardiac disease patient population, including intolerance to ADDs leading to low medication adherence [3], increased risk of adverse cardiovascular events [53–55], and limited resources for delivery of CBT and similar behavioral therapies [56, 57]. Thus, there is a pressing need for interventions specifically designed to reduce depressive symptoms in older cardiac patients with StD.

Our subliminal priming with supraliminal reward stimulation (SPSRS) website may provide effective intervention by displaying positive word stimuli embedded in videos [58]. A major advantage of SPSRS is free unlimited access, allowing use anytime, anywhere, and for any duration. Moreover, SPSRS uses the YouTube application programming interface, so patients can choose from a variety of videos according to personal interest. Further, there is no limit on the number of viewers or individual views. Previous studies support the potential efficacy of SPSRS for people with StDs without adverse events as this intervention requires only passive viewing (e.g., while lying down or sitting) [59–63]. Therefore, SPSRS is safe and suitable for both outpatient and in-hospital use.

However, randomized controlled trials (RCTs) are needed to investigate and validate the efficacy of SPSRS interventions in older cardiac patients with StD. Therefore, the Medical Research Council methodological framework (including development, feasibility and piloting, evaluation, and implementation phases) was used to test the efficacy of viewing videos with positive word stimuli using the SPSRS on depressive symptoms in older cardiac patients with StD [64, 65]. The current study is a pilot RCT to evaluate the feasibility of implementing a full-scale RCT. We hypothesize that older cardiac patients with StD who receive the SPSRS intervention will show greater improvement in depressive symptoms after treatment than controls viewing similar videos without embedded positive words.

## Methods

### Trial design

This study was designed as a 1-week, single center, open-label, pilot, randomized, parallel-group trial testing the antidepressant efficacy and feasibility of the SPSRS intervention, and was conducted with the approval of the Ethical Review Board for Medical Research Involving Human Subjects of Gunma University (approval number: HS2023-050) and the Ethical Review Committee of Fujioka General Hospital (Approval No. 338). Further details on the study design are provided in the protocol paper [66]. The study adhered to CONSORT2010 guidelines for randomized pilot and feasibility studies [67].

### Participants

Candidates for this study were recruited from the acute care ward of Fujioka General Hospital, Gunma, Japan. The participants were selected according to the following criteria: patients of either sex admitted to acute care wards of Fujioka General Hospital, aged 65 years or older, with diagnosed cardiac disease (coronary artery disease, heart failure, heart valve disease, and arrhythmia), scoring 10 or more on the Japanese version of the Beck Depression Inventory-II (BDI-II) [68], sufficient cognitive ability to understand the content and purpose of the research (according to the Japanese Mini-mental State Examination, [MMSE-J]), New York Heart Association (NYHA) functional classification I–III, and willing to provide written informed consent. Candidates were excluded for past or current psychiatric disorders, professional treatment for mental health issues, visual or hearing impairments that interfere with daily living, a major depressive episode within the previous 2 weeks as assessed by the Mini-international Neuropsychiatric Interview (M.I.N.I.) [69, 70], and life-threatening complications (such as severe organ failure, respiratory disorders, cerebrovascular disorders, and musculoskeletal disorders).

Generally, the cutoff score for the BDI-II is ≥ 14 points [71]; however, some studies have indicated that the cutoff score for using the BDI-II to determine the presence of StD is ≥ 10 points [68]. Therefore, in this study, the cutoff score for the BDI-II was set at ≥ 10 points. Furthermore, in this study, no clear cutoff value was set for the MMSE-J, and the researchers determined the presence or absence of cognitive function in the participants on the basis of the MMSE-J score, as well as the responses to each item and the patient’s response to verbal instructions during the assessment [72]. Previous studies have suggested that patients with moderate-to-severe cognitive impairment can still make informed decisions regarding their treatment plans and ethics, and cognitive function for study participation should not be based solely on the MMSE-J cutoff score[73–75].

### Settings and recruitment

Fujioka General Hospital is a 399-bed public hospital specializing in the treatment and rehabilitation of disuse syndrome, musculoskeletal disorders, cerebrovascular disorders, respiratory disorders, cardiovascular disorders, cancer, and pediatric disorders. The acute care ward provides individual therapy by physical therapists (PTs), occupational therapists (OTs), and speech therapists (STs) for up to 2–3 h each day, 7 days a week, 365 days a year to promote independence in activities of daily living and discharge home. All patients in this acute care ward are assigned a specific PT, OT, and (or) ST as required, although an alternate may provide rehabilitation when the assigned therapist is absent or unable to intervene. The participants were given a brochure about the study within a week of admission and encouraged to participate. The eligibility evaluation was conducted within 3 days of obtaining informed written consent. After the baseline assessment, the participants were randomly divided 1:1 into an intervention group (video viewing on the SPSRS website with positive word stimuli, *n* = 15) and a control group (video viewing on YouTube without positive word stimuli, *n* = 15). Details on the recruitment material are provided in the protocol paper [66].

### Interventions

Participants in both groups watched a 10-min video once each day for 5 consecutive days (at least 50 min in total) on an iPad at the bedside in the sitting or lying position and in the company of a researcher. The SPSRS is similar to a general motion playback website, allowing users to find and watch videos by keyword searches except that SPSRS videos display positive language stimuli starting with the words “can,” “let us try,” “good luck,” “able,” and “do not worry” at random in the four corners of the video screen for 17 ms at 5-s intervals and then the positive words “nice,” “great,” “fantastic,” “satisfactory,” and “enjoyable” at the center of the video screen for 150 ms at 5-s intervals [76, 77]. The SPSRS website was operated by the researchers, and the participants selected and watched videos of interest to them. Alternatively, the control group viewed videos of choice on YouTube for the same durations.

### Assessment measures

Age, sex, primary disease, comorbid diseases, Mini-mental State Examination (MMSE)-Japanese score [78, 79], left ventricular ejection fraction, and blood laboratory results (serum albumin, hemoglobin, creatinine, and brain natriuretic peptide levels) were recorded at baseline.

### Primary outcome measure

The primary outcome was the change in BDI-II (Japanese) score from baseline to postintervention 1 week later. The BDI-II is a 21-item self-administered questionnaire that measures the severity of depressive symptoms [80]. Items are rated using a four-point Likert scale ranging from 0 to 3 points, yielding total scores from 0 to 63 points, with higher total scores indicating more severe depressive symptoms. The reliability and validity of the BDI-II (Japanese version) have been reported elsewhere [80–82].

### Secondary outcome measures

#### New York heart association functional classification

Participants were also stratified according to the NYHA functional classification based on the degree of subjective symptoms caused by physical activity. Briefly, heart failure severity was classified as follows: Grade I (presence of cardiac disease but no limitation of physical activity), Grade II (mild to moderate limitation of physical activity on exertion, asymptomatic at rest), Grade III (severe limitation of physical activity on exertion, asymptomatic at rest), and Grade IV (cardiac disease that limits any physical activity with heart failure symptoms and anginal pain even at rest) [83].

#### Specific activity scale (SAS)

The specific activity scale (SAS) measures the estimated minimum intensity of exercise (oxygen uptake or metabolic equivalents [METs]) conferring limitations in activities of daily living. Briefly, the patients are asked to answer “Yes,” “No,” or “unknown” in sequence starting from the lowest physical activity level. If a given level is answered “Yes,” the question is re-posed for higher activity, and the point at which the respondent answers “No” is recorded as the minimum exercise intensity (Met) at which heart failure symptoms appear. A higher calculated Met is thus indicative of greater exercise tolerance. The validity and reliability of the SAS have been reported [84, 85].

#### Grip strength

Grip strength was measured using a digital dynamometer (TKK 5401 GRIP-D; Takei, Japan) in the sitting position, with the shoulder joint at 0° and the elbow joint in extension [86, 87]. The measurements were conducted twice on each side and the maximum value included in the analysis. It has been suggested that grip strength reliably correlates with overall muscle strength [88].

#### Blinding

Blinding of participants and researchers was not possible due to differences in viewing platform and the small number of research staff participating in the study. Therefore, the study was “open-label.” However, baseline assessments were performed prior to randomization. In addition, primary and secondary outcomes were measured in a standardized manner. Thus, the potential for bias was minimized.

#### Sample size calculation

As there are no previous studies assessing the antidepressant efficacy of this SPSRS interventions for older cardiac patients with StD hospitalized in acute care wards, a formal sample size calculation was not conducted. However, a sample of 15–20 participants per group is considered acceptable for a pilot study [89, 90], so a total of 30 participants meeting inclusion and exclusion criteria was recruited.

#### Randomization

Participants meeting eligibility criteria were randomly assigned to the intervention or control group following baseline assessments. An independent third-party created a randomized list based on the permuted block method (block size 2) in an Excel spreadsheet. This randomized list was sent to a central registry center at Gunma University to avoid selection bias during the assignment.

### Statistical analysis

The primary and secondary outcome measures post-intervention were compared to baseline measures by restricted maximum likelihood estimation using linear mixed models (LMMs). In these models, BDI-II score (primary outcome) was set as the dependent variable, group (intervention vs. control), time (baseline vs. post-intervention), and group × time interaction as fixed effects, and participants as random effects. A two-tailed *p* < 0.05 was considered statistically significant for both the primary and secondary outcomes. All statistical analyses were performed using IBM SPSS v.30.0.0.0 (IBM Japan, Tokyo, Japan). In addition, Hedge’s *g* was calculated as an indicator of the group difference in outcome change following the intervention [91, 92], with g of 0.2 considered a small, g of 0.5 a moderate, and g of 0.8 a large difference [93].

## Results

### Enrollment and baseline characteristics

**Fig. 1:**
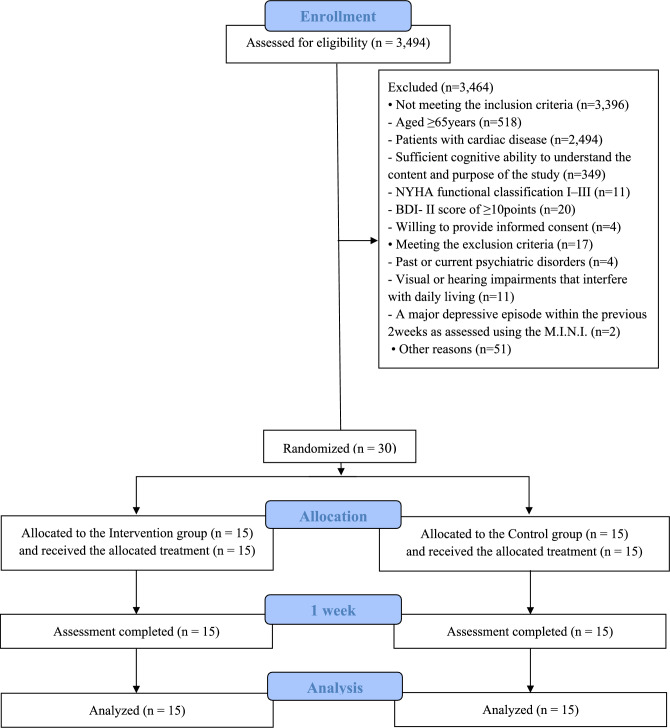
Trial flowchart

Figure 1 presents the trial flowchart. Between October 2023 and October 2024, 3494 candidates were assessed for eligibility and 30 meeting inclusion and exclusion criteria ultimately selected as participants. After baseline assessments, participants were randomly assigned to the intervention group (*n* = 15) or the control group (*n* = 15). No participants dropped out during the study. Table 1 summarizes the baseline demographic parameters of both groups. There were no significant group differences in mean age, sex ratio, MMSE-J score, LVEF, blood chemistry parameters, and disease subtype distribution.

**Table 1:** Baseline characteristics of the SPSRS intervention and control groups

	Total (*n* = 30)	Intervention group (*n* = 15)	Control group (*n* = 15)
Characteristics			
Age (years)	79.2 (7.55)	81.33 (7.82)	77.07 (6.88)
Sex			
Female	6 (20.0)	3 (20.0)	3 (20.0)
MMSE-J	26.5 (2.4)	25.80 (2.34)	27.13 (2.42)
LVEF (Teichholz method)	41.4 (13.9)	44.13 (14.85)	38.60 (12.68)
ALB (g/dl)	3.9 (0.35)	3.90 (0.27)	3.85 (0.42)
Hgb (g/dl)	12.1 (1.9)	11.98 (1.56)	12.18 (2.30)
CRE (mg/dl)	1.39 (1.08)	1.21 (0.50)	1.56 (1.45)
BNP (pg/ml)	862.1 (874.0)	721.83 (799.18)	1002.43 (949.49)
Diagnosis			
Heart failure	17 (56.7)	9 (60.0)	8 (53.3)
Coronary artery disease	11 (36.7)	6 (40.0)	5 (33.3)
Heart valve disease	1 (3.3)	0 (0)	1 (6.7)
Arrhythmia	1 (3.3)	0 (0)	1 (6.7)
Admission diagnosis			
Hypertension	9 (30.0)	4 (26.7)	5 (33.3)
Diabetes	9 (30.0)	2 (13.3)	7 (46.7)
Heart failure	7 (23.3)	5 (33.3)	2 (13.3)
Coronary artery disease	5 (16.7)	2 (13.3)	3 (20.0)
Heart valve disease	5 (16.7)	2 (13.3)	3 (20.0)
Arrhythmia	15 (50.0)	9 (60.0)	6 (40.0)

Data presented as mean (standard deviation) or number (%)

*MMSE-J* Mini-mental State Examination-Japanese, *LVEF* Left ventricular ejection fraction, *ALB* Albumin, *Hgb* Hemoglobin, *CRE* Creatinine, *BNP* Brain natriuretic peptide

### Impact of the SPSRS intervention on outcomes

Table 2 presents the mean, SD, LMM, and Hedge’s g values for the measured outcomes at baseline and post-intervention for SPSRS and control groups. Mean BDI-II scores did not differ significantly between groups (SPSRS vs. control, *p* = 0.43) or evaluation times (baseline vs. post-intervention, *p* = 0.06), and there was no group × time interaction (*p* = 0.72). Further, the group difference was small according to Hedge’s g value (*g* = 0.13). Mean NYHA functional score differed between evaluation times (*p* = 0.02) but not groups (*p* = 0.80), and there was no significant group × time interaction (*p* = 0.81). Mean SAS score also differed between evaluation times (*p* = 0.01), but again not between groups (*p* = 0.27), and there was no group × time interaction (*p* = 0.43). Hedge’s g was also small (*g* = 0.09). Hedge’s g was − 0.46. Finally, grip strength did not differ between groups (*p* = 0.86) or measurement times (*p* = 0.51), and there was no significant group × time interactions (*p* = 0.05). However, Hedge’s g value was moderate (*g* = 0.72).

**Table 2:** Results of LMM and effect size analyses

	Intervention group (*n* = 15)	Control group (*n* = 15)	LMM				Standardized mean difference	
Outcomes	Mean (SD)	Mean (SD)	Effect	Estimate	P	95% CI	Hedges’ g	95% CI
BDI								
Pre-	18.53(3.48)	16.40 (8.24)	Group	− 2.13	0.43	− 7.576 to 3.309	− 0.13	− 0.83 to 0.57
Post-	15.53 (7.67)	12.60 (8.84)	Time	− 3.00	0.06	− 6.155 to 0.155		
Average change	3 (6.32)	3.80 (5.58)	Interaction	− 0.80	0.72	− 5.262 to 3.662		
NYHA								
Pre-	2.33(0.62)	2.27 (0.70)	Group	− 0.07	0.80	− 0.584 to 0.450	0.09	− 0.60 to 0.79
Post-	1.87 (0.64)	1.87 (0.83)	Time	− 0.47	0.02	− 0.858 to − 0.075		
Average change	0.47 (0.52)	0.40 (0.91)	Interaction	0.07	0.81	− 0.487 to 0.620		
SAS								
Pre-	3.43(1.37)	4.13(1.58)	Group	0.70	0.27	− 0.571 to 1.971	− 0.46	1.76 to 0.84
Post-	4.50 (1.70)	4.77 (2.15)	Time	1.07	0.01	0.275 to 1.858		
Average change	− 1.01 (1.46)	− 0.63 (1.53)	Interaction	− 0.43	0.43	− 1.552 to 0.686		
Grip strength								
Pre-	25.00 (9.31)	25.55 (7.90)	Group	0.55	0.86	− 5.843 to 6.949	0.72	0.00 to 1.44
Post-	25.43 (8.82)	24.15 (8.16)	Time	0.43	0.51	− 0.889 to 1.742		
Average change	0.43 (1.86)	− 1.4 (2.98)	Interaction	− 1.83	0.05	− 3.687 to 0.034		

*LMM* Linear mixed model, *BDI-II* Beck Depression Inventory-II, *NYHA* New York Heart Association functional classification, *SAS* Specific Activity Scale

## Discussion

This pilot RCT provided valuable insights into the feasibility of using the SPSRS intervention for depressive symptoms in older patients with cardiac disease and StD. Notably, this trial revealed no study participation-associated adverse events in the experimental and control groups. This finding suggests that the SPSRS intervention can be safely employed to older patients with cardiac disease and StD who are hospitalized in the acute care ward. Furthermore, as the trial had no dropouts, the data were collected from all registered participants, and no data management issues were observed. This fact suggests the possibility of conducting a full-scale RCT of the SPSRS intervention for depressive symptoms in older patients with cardiac disease and StD.

The feasibility of using the SPSRS intervention for depressive symptoms in older patients with cardiac disease and StD may be enhanced by synthesizing the cumulative results of this trial and implementing adjustments to ensure the success of future full-scale RCTs. First, the recruitment process could be streamlined. To achieve the target sample size (*n* = 30) for this study, a large number of participants should be recruited. Specifically, 3494 potential participants should be screened between October 2023 and October 2024, majority of whom did not have cardiac disease (e.g., coronary artery disease, heart failure, heart valve disease, and arrhythmia). As the majority of the reasons for the early termination of RCTs are due to the lack of participants in the recruitment process and that future full-scale RCTs require larger sample sizes, strategies for recruiting potential participants should be considered. A multicenter RCT recruiting a larger and more diverse candidate pool represents a realistic and effective solution. Therefore, establishing additional procedures for efficient communication between the study teams is necessary.

Second, the SPSRS intervention could be modified. We hypothesized that the SPSRS video intervention would be more effective than a regular video intervention (YouTube video viewing) for managing depressive symptoms in older patients with cardiac disease and StD. However, contrary to our hypothesis, the SPSRS intervention did not significantly improve the depressive symptoms of older patients with cardiac disease and StD compared with YouTube video viewing, and the effect of the SPSRS intervention was negligible. Therefore, the SPSRS intervention may be ineffective for the depressive symptoms of older patients with cardiac disease and StD. However, this trial was conducted with the smallest sample size necessary for a pilot RCT [90]. Consequently, the estimated precision was low, and accurately estimating the effect of the SPSRS intervention on depressive symptoms in older patients with cardiac disease and StD on the basis of the results of this trial was challenging. In fact, the 95% CI for the LMM analysis of this study was relatively large. However, several factors may explain why the SPSRS video intervention was not more effective than the usual video intervention (YouTube video viewing) for depressive symptoms in older patients with cardiac disease and StD. The first factor may be the intervention time of this study. In a previous RCT conducted on individuals with StD, participants watched videos for 10 min/day for 5 weeks. Consequently, it was suggested that SPSRS moderately improves the depressive symptoms of individuals with StD [59]. Conversely, the participants of this trial encompassed older patients with cardiac disease and StD and were being treated in an acute care ward. Therefore, along with the short hospital stay and the fact that implementing the 5-week SPSRS intervention may be difficult, the results of previous studies that suggested the possibility of significantly improving the depressive symptoms of patients through a 1-week intervention were considered, and the intervention period was set at 1 week in this study [94, 95]. In this trial, the differences in the intervention period duration and the length of time spent watching the videos on the SPSRS may explain why the SPSRS intervention did not reduce the depressive symptoms of older patients with cardiac disease and StD. Therefore, future full-scale RCTs may need to consider adjusting the intervention schedule (e.g., watching 10-min videos twice daily).

The second factor may be that although the BDI-II is widely used for assessing clinical populations, including patients with cardiac disease [94–97], this evaluation encompasses the previous 2 weeks and may not be sufficiently sensitive to detect changes incurred over 1 week. Therefore, future studies should select tests measuring depressive symptoms over shorter periods, including the Quick Inventory of Depressive Symptomatology or the Center for Epidemiologic Studies Depression Scale (CES-D) [98–102]. Although the CES-D and BDI-II measure several domains of depressive symptoms, the CES-D yields a four-factor solution comprising Depressed Affect, Positive Affect, Somatic and Retarded Activity, and Interpersonal Troubles, whereas Positive Affect is not included in the BDI-II or Patient Health Questionnaire-9. Considering that the SPSRS uses positive language stimuli for improving subthreshold depressive symptoms, the CES-D score may be a more suitable evaluation scale. In fact, a previous RCT on patients with StD employed the CES-D for depressive symptom evaluation [59, 103, 104]. Therefore, future full-scale RCTs should employ scales that assess specific aspects of depressive symptoms, including the CES-D and Quick Inventory of Depressive Symptomatology, as they could reveal antidepressant efficacy even after shorter intervention periods.

The third potential modification is altering the words displayed (SPSRS content). The positive word stimuli displayed were identified through qualitative research conducted on adolescents with StD [76, 77]; therefore, these words are likely not as effective in older adults. Evaluating other words for their effects on confidence and mood in older patients with cardiac disease and StD may be necessary.

In summary, to improve the feasibility and power of a full-scale RCT on the antidepressant efficacy of the SPSRS in older patients with cardiac disease and StD, the sample size should be increased (possibly by recruiting at multiple institutions), the daily or weekly exposure duration prolonged, multidimensional evaluation outcomes used in place or in addition to the BDI-II, and stimuli selected with demonstrated effects on confidence and mood in this specific patient group.

No significant group differences were noted in terms of secondary outcomes (NYHA and SAS), and the effect sizes were small. In this study, these measures were adopted as secondary outcomes because they are widely used in clinical and study settings for evaluating the exercise endurance of patients with cardiac disease [83–85]. However, these scales may not accurately capture the typical functional status of older patients with cardiac disease and StD. Therefore, when conducting a full-scale RCT, adopting a different scale for measuring exercise tolerance, including the 6-min walk test, may be necessary [105]. Another possible reason is that the short 1-week SPSRS intervention may have been insufficient to yield significant improvements in objective and subjective exercise tolerance. Considering that cardiac diseases and StDs have a synergistic effect on risk, adjusting the viewing time of SPSRS (e.g., watching 10-min videos twice daily) and causing an improvement in depressive symptoms may improve objective or subjective exercise tolerance.

Furthermore, no significant difference in grip strength was observed between the two groups; however, surprisingly, the SPSRS intervention caused a moderate numerical improvement compared with the control group, in accordance with previous word stimulation studies [77, 106, 107]. This improvement in grip strength suggests that the SPSRS intervention can enhance exercise tolerance among older patients with cardiac disease and StD. Previous studies have suggested that decreased grip strength is prevalent among older patients with cardiac disease and is associated with more severe depressive symptoms [108–110]. Therefore, in older patients with cardiac disease, the effects of SPSRS may manifest as improved grip strength, potentially leading to reduced depression severity. However, in this study, the 95% CIs for the LMM analysis (− 3.687 to 0.034) and Hedges’ g (0.00–1.44) were relatively large. Therefore, future full-scale RCTs may need to investigate the effect of the SPSRS intervention on grip strength in older patients with cardiac disease and StD using a larger sample size.

This trial had several limitations. First, the single-center design may introduce selection bias and reduce the generalizability of the findings despite the high external validity conferred by the inclusion of only older patients with cardiac disease and StD in an acute care ward [111]. Second, owing to the nature of the trial, it was conducted as an open-label study. Consequently, other factors, including patient expectations and effects on the therapist–patient relationship, may have impacted depressive symptoms. Third, the sample size was set to a minimum and formal sample size calculations could not be performed, thereby limiting the statistical power for detecting differences in primary and secondary outcomes. Fourth, this study was conducted on older patients with cardiac disease and StD who were hospitalized in an acute care ward. Therefore, the effects on other groups were unknown. A previous study suggested that SPSRS interventions can improve depressive symptoms in community-dwelling individuals with StDs [59]. Therefore, investigating the effects of SPSRS interventions on, for example, older adults with cardiac disease and StDs receiving outpatient care, may be required. Finally, the videos viewed were self-selected; therefore, individual differences in the video content may have enhanced the response heterogeneity and diminished the detectability of the effects on depressive symptoms.

## Conclusions

This is the first pilot RCT to examine the feasibility of an SPSRS intervention for older patients with cardiac disease and StD hospitalized in an acute care ward. The results of this pilot RCT indicate that several modifications are required before full-scale trials are warranted. Nonetheless, these results demonstrate the potential limitations of SPSRS for managing depressive symptoms in this specific population.

## Data Availability

Data are available from the corresponding author on reasonable request.
